# Mesenchymal stem cells and platelet-rich plasma-impregnated polycaprolactone-β tricalcium phosphate bio-scaffold enhanced bone regeneration around dental implants

**DOI:** 10.1186/s40729-021-00317-y

**Published:** 2021-05-05

**Authors:** Akram Abdo Almansoori, Oh-Jun Kwon, Jeong-Hun Nam, Young-Kwon Seo, Hae-Ryong Song, Jong-Ho Lee

**Affiliations:** 1 Department of Oral & Maxillofacial Surgery, School of Dentistry, Seoul National University, Seoul, Korea; 2 Craniofacial Tissue Engineering and Stem Cells, Faculty of Dentistry, McGill University, Montreal, Canada; 3 Department of Chemical and Biochemical Engineering, College of Engineering, Dongkuk University, Seoul, Korea; 4 Department of Orthopedics and Dwarfism Clinic, Korea University Guro Hospital, Seoul, Korea; 5 Innovation Research and Support Center for Dental Science, Seoul National University Dental Hospital, Seoul, Korea

**Keywords:** Mesenchymal stem cells, Platelet-rich plasma, Polycaprolactone, β-Tricalcium phosphate, Guided bone regeneration, Dental implants

## Abstract

**Background:**

Finding a material that supports bone regeneration is the concern for many investigators. We supposed that a composite scaffold of poly(ε) caprolactone and β-tricalcium phosphate (PCL-TCP) would entail desirable characteristics of biocompatibility, bioresorbability, rigidity, and osteoconductivity for a proper guided bone regeneration. Furthermore, the incorporation of mesenchymal stem cells (MSCs) and platelet-rich plasma (PRP) would boost the bone regeneration. We conducted this study to evaluate the bone regeneration capacity of PCL-TCP scaffold that is loaded with MSCs and PRP.

**Materials and methods:**

Five miniature pigs received 6 implants in 6 created-mandibular bony defects in the right and left lower premolar areas. The bony defects were managed according to the following three groups: the PCL-TCP scaffold loaded with MSCs and PRP (MSCs+PRP+PCL-TCP) group (*n* = 10), PCL-TCP scaffold loaded with PRP (PRP+PCL-TCP) group (*n* = 10), and PCL-TCP scaffold group (*n* = 10). After 12 weeks, the bone regeneration was assessed using fluorochrome bone labeling, μCT bone morphogenic analysis, and histomorphometric analysis.

**Results:**

All of the three groups supported the bone regeneration around the dental implants. However, the PCL-TCP scaffold loaded with MSCs and PRP (MSCs+PRP+PCL-TCP) group showed non-significant higher bone surface, bone specific surface, and bone surface density than the other two groups as revealed by the μCT bone morphogenic analysis. Histologically, the same group revealed higher bone-implant contact ratio (BIC) (*p* = 0.017) and new bone height formation (NBH, mm) (*p* = 0.0097) with statistically significant difference compared to the PCL-TCP scaffold group.

**Conclusions:**

PCL-TCP scaffold is compatible for bone regeneration in bone defects surrounding dental implants. Moreover, the incorporation of MSCs and PRP optimized the bone regeneration process with respect to the rate of scaffold replacement, the height of the regenerated bone, and implant stability.

## Background

Achieving proper dental implant stability and survivability has been challenged by the deficiency of the hosting alveolar bone in respect to height, width, and well-maintained bony walls [1]. Autologous bone graft has been the standard approach to reconstruct the alveolar bony defects due to its osseoinductive, osseoconductive, and osteogenic properties. Nevertheless, the high resorption rate of the autologous bone grafts, which could reach 60%, may jeopardize the clinical outcomes [2]. Moreover, the autologous bone graft might be associated with donor site morbidity such as, infection, pain, blood loss, and scarring [3].

Recently, guided bone regeneration has grown immensely. This procedure includes the application of a membrane to exclude non-osteogenic tissue from interfering with bone regeneration. This membrane can be resorbable or non-resorbable and it should have some essential properties like biocompatibility, clinical manageability, and space making ability [4]. Polycaprolactone (PCL)-based scaffolds are characterized by biocompatibility, bioresorbability, and manageability. However, they lack rigidity, stability, and osteoconductive capacity [5]. The incorporation of β-tricalcium phosphate (TCP) had enhanced the rigidity and osteoconductivity of PCL based scaffolds [6]. In terms of bone regeneration, mesenchymal stem cells (MSCs) and platelet-rich plasma (PRP) have been reported to boost the bone formation through promoting the osteoblasts formation and vascularization [7–9]. Accordingly, we conducted this study to evaluate the bone regeneration capacity of PCL-TCP scaffolds loaded with MSCs+PRP within peri-implant bony defects.

## Materials and methods

### Study design

The animal studies were performed after receiving approval of the Institutional Animal Care and Use Committee (IACUC) in Seoul National University. Five male miniature pigs (SPF Micropig®, PWG Genetics Korea, Ltd., Korea), aged 12.5 to 17.5 months (32.8 to 41.0 kg in weight), were used in this study. Each miniature pig received 6 implants; 3 implants on each side of the inferior bone of the mandible at the premolar area. Buccal bony defects were created after the implant site preparation and before the implants placement. A total of 30 buccal bone defects were equally distributed to 10 bony defects for each of the designed three groups (2 out of 6 defects assigned for each of the three groups in each animal):

- *MSCs+PRP+PCL-TCP group*. Mesenchymal stem cells and platelet-rich plasma loaded into polycaprolactone - β-tricalcium phosphate scaffold (*n* = 10)
- *PRP+PCL-TCP group*. Platelet-rich plasma loaded into polycaprolactone-β-tricalcium phosphate scaffold (*n* = 10)
- *PCL-TCP group*. Polycaprolactone-β-tricalcium phosphate scaffold (*n* = 10)

### PCL-TCP scaffold fabrication

**Fig. 1:**
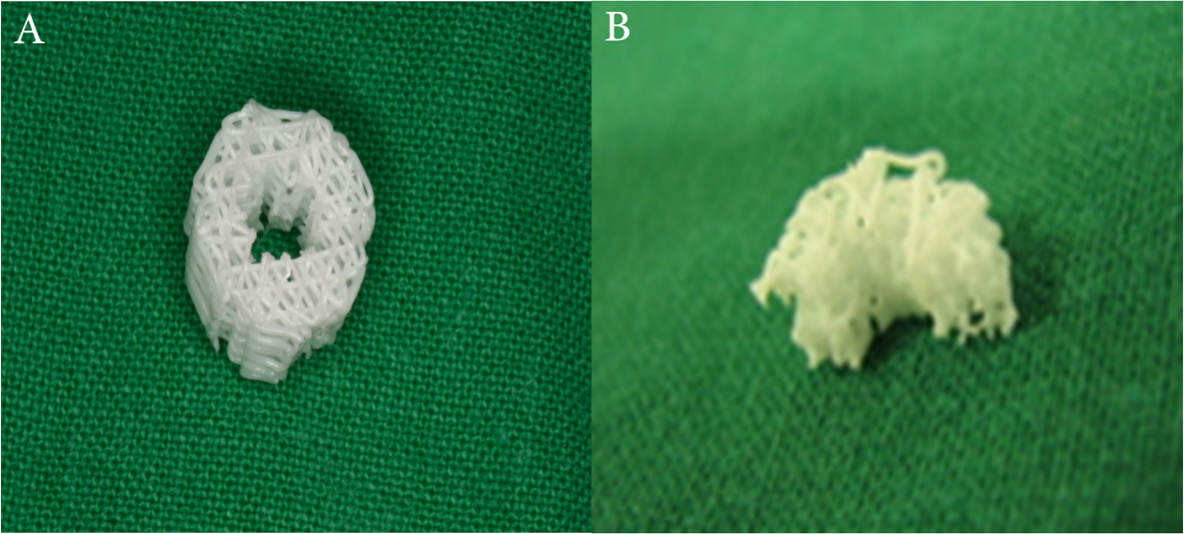
The PCL-TCP scaffold structure. **a** Tube-shape scaffold with 4-mm inner diameter, 8-mm outer diameter, 4-mm height, and 2-mm thickness. **b** The scaffold was cut in half to fit the created buccal mandibular bony defects

A composite scaffold of PCL-TCP (80:20%) (Osteopore International Pte. Ltd., Singapore) was fabricated using the fused deposition modeling method that was described in previous reports [10, 11]. The scaffold was designed as honeycomb structure with 0°, 60°, and 120° cross linking; 70% porosity and 0.515 mm average pore size. The scaffold was cut into a half-tube shape with 4-mm inner diameter, 8-mm outer diameter, and 2-mm thickness. This dimension was selected to allow the scaffold to fit around the dental implants promptly (Fig. 1).

### Human umbilical cord derived mesenchymal stem cells (hUCMSCs)

The cells were obtained from the Research Institute of Biotechnology at Dongguk University, Korea. The cells were found positive against CD 73, CD 90, CD 105, and CD 166 markers and negative against CD14, CD 31, and CD 45 markers using fluorescence activated cell sorting (FACS) analysis. The cells differentiation abilities were proved by inducing the cells to differentiate into osteocytes, chondrocytes, and adipocytes.

The cells were seeded and expanded using low glucose Dulbecco’s modified Eagle’s medium (LG-DMEM; Gibco-BRL, USA) containing 10% fetal bovine serum (FBS; Gibco-BRL, USA) and 1% penicillin/streptomycin (PAA laboratories, Germany). Cells medium was changed every 3 days. The cells were harvested at 80% confluency and 5 passages and then loaded into the scaffold of MSCs+PRP+PCL-TCP group with cell density of 4 × 10^7^ cells/ml at the day of implantation using a micropipette.

### Platelet-rich plasma (PRP) preparation

PRP preparation and loading took place on the day of operation at the time of the dental implants sites preparation. A total of 200 ml of blood was aspirated and divided into 4 tubes with 50 ml per each. The tubes were already coated with 0.2 gm EDTA as anticoagulant. The PRP preparation conducted in two-steps. The blood was first centrifuged at 3200 rpm for 5 min and the middle buffy coat and part of the plasma were aspirated into new tube where they were centrifuged at 5200 for 5 min. The result lower middle PRP was aspirated and kept into a sample shaker for 30 min at room temperature. Every 50 ml of blood resulted in 1.0~1.5 ml of PRP. Reagents of thrombin and CaCl_2_ (1:1) (Thrombin, 1000 units, Dirabine® powder, Korea united pharm., Co., Korea) (CaCl_2_, 100 mg/mL, Calmia® Inj., Korea united pharm., Co., Korea) were mixed with the PRP at 1:6 volume ratio for creating an activated gel form. A 0.2~0.5 ml of that PRP gel was loaded into each scaffold of MSCs+PRP+PCL-TCP group and PRP+PCL-TCP group.

### Surgical procedure

**Fig. 2:**
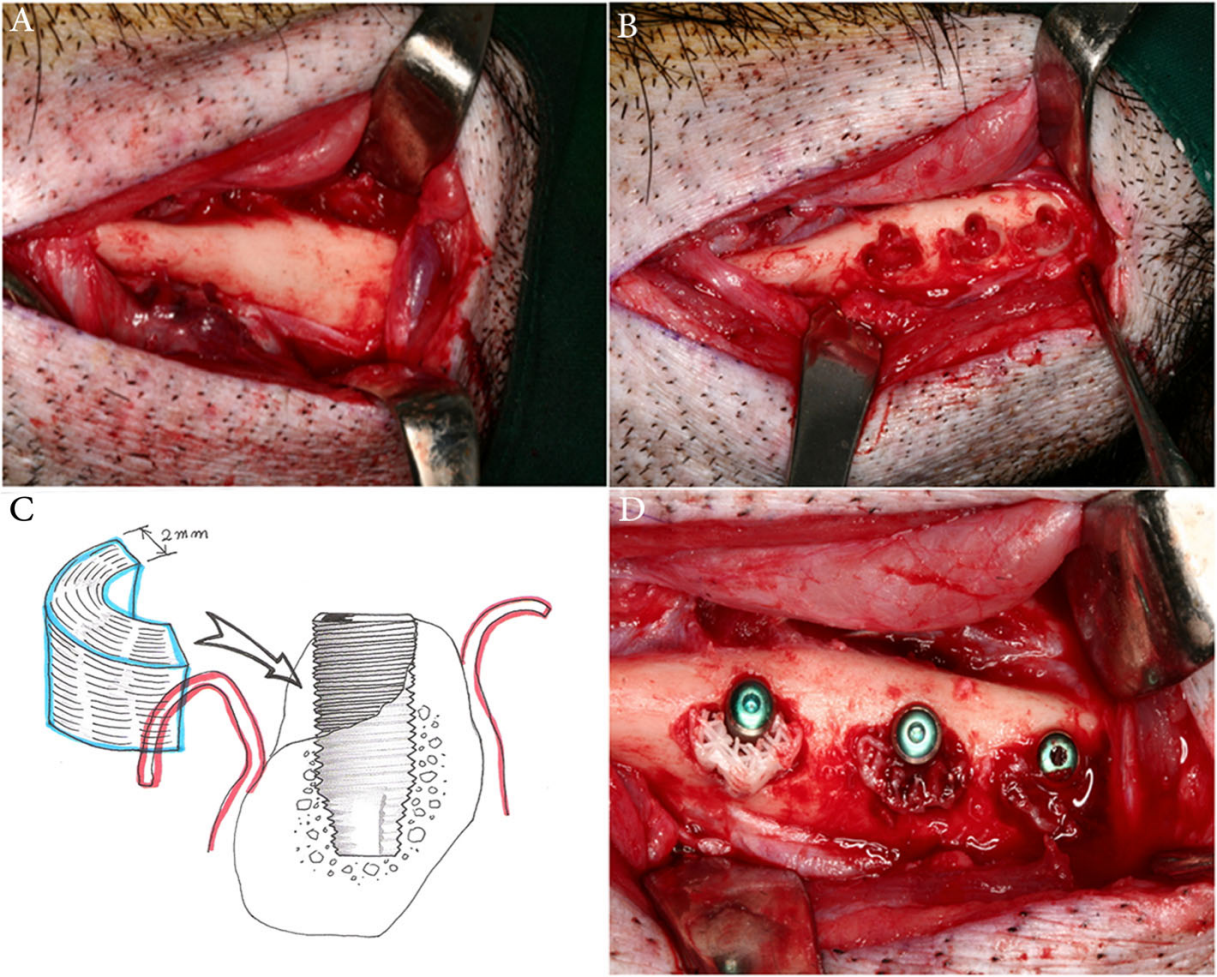
Intraoperative photographs showing the implants site drilling and placement of the MSCs-PRP-loaded PCL-TCP scaffolds. **a** Incision was made along the inferior border of the mandible bilaterally within the premolar area. **b** Three implant sites were drilled with buccal half circle bony defects. **c**, **d** The implants were placed and the scaffolds were placed to cover them

General anesthesia was induced with an intra-muscular (IM) injection of zoletil and xylazine and maintained with isoflurane inhalation (0.5–2%). The lower face was shaved, swapped with iodine solution, and draped sterile. The mandibular inferior border was incised bilaterally within the borders of the premolar areas (Fig. 2a). Subperiosteal flaps were reflected, the implants sites were prepared in the proper sequence, and half-round bony defects were created buccally with diameter of 8 mm, depth of 2 mm, and height of 4 mm (Fig. 2b). Thereafter, bone-level implants (Avana^Ⓡ^, GSII, Osstem Co., Korea) with a length of 8.5 mm and a diameter of 4.0 mm were inserted and the defects were filled with the customized scaffolds (Fig. 2c, d). The stem cells were seeded into the PCL-TCP scaffold (4.0 × 10^6^ cells/scaffold) by a drop down and soaking method using a micropipette and the prepared PRP was loaded into the scaffold (0.2~0.5 ml/scaffold). The flaps were repositioned and closed in layers. Intra-muscular injections of oxytetracycline 3 ml and flunixin meglumine 2 ml were given for 3 days post-operatively.

### Post-operative CT scan

Three days after the operation, a mandible CT scan was taken to confirm the implant-scaffold stability and rule out any post-operative complications.

### Fluorochrome bone labeling

To observe the histological sequence of bone regeneration, three fluorochromes were intra-muscularly injected at frequent times within 12 weeks post-operatively. Tetracycline HCL 15 mg/kg was administered at 3 and 11 weeks. Calcein (10 mg/kg) and alizarin red (30 mg/kg) fluorochromes were administered at 6 and 9 weeks, respectively. The animals were sacrificed after 12 weeks. The colorful regenerated bands were checked using a confocal laser scanning microscope (Olympus-Flouview300, Olympus Co., Tokyo, Japan). The color filter ranges were red 530–560/580 nm for red alizarin, green 494/517 nm for calcein, and yellow 525–560 nm for tetracycline.

### Micro-CT bone morphogenic analysis

Using 5 implant-grafted sites, micro-CT images were obtained using X-ray microtomography system (Skyscan 1072/1172, ver. 1.5, Belgium) under the settings of × 14 magnification, 80 kV/100 mA, 180° rotation, 0.90° rotation step, 3.4-s exposure time, and no filtering. The reference Hounsfield unit (HU) was used to assure optimal digital images with minimal beam hardening effects (0.7000 HU to 0.1000 HU values in 1072 system and 0.1100 HU to 0.0030 HU in 1172 system). The digitalized bitmap image files were analyzed using CTAn® software program (Skyscan Co., Belgium). The region of interest (ROI) was drawn over the implant and scaffold within 0.5 mm lateral to their borders. The 3-D analysis data of the reference PCL-TCP scaffold was 255.12 mm^3^ in tissue volume (TV) and 240.29 mm^2^ in bone surface (BS). The new bone formation capacity was analyzed using the morphometric parameters of total tissue volume (TV, mm^3^), bone volume (BV, mm^3^), bone volume fraction ratio (BV/TV, %), regenerative new bone surface area (BS, mm^2^), bone specific surface (BS/BV, mm^−1^), bone surface density (BS/TV, mm^−1^), and interception surface (I.S, mm^2^). Three-dimensional rendered models were obtained using the Vworks® software program (Osstem Co., Korea).

### Histomorphometric analysis

Five implant-scaffold non-decalcified bone blacks were embedded in light curing resin (Technovit 7200 VLC+BPO, Kulzer & Co., Germany). They were then cut longitudinally parallel to the long axis of the implants using the Exact Cutting and Grinding Equipment (Exact Apparatebau, Norderstedt, Germany) into 30-μm-thick sections. Thereafter, the samples were stained using hematoxylin-eosin (H&E) and Masson’s trichrome (MT) staining. New bone height (NBH, mm), new bone area (NBA, mm^2^), bone-implant contact (BIC) ratio, and residual scaffold areas (RSA, mm^2^) were measured in the experimental sites using Kappa Imagebase® (Kappa optoelectronics**,** Germany) software.

### Statistics

All data were presented as mean ± standard error. Values of *p* < 0.05 were considered to be statistically significant. Repeated measures ANOVA were used for consecutive comparisons among each of the groups. One way ANOVA was used for multiple comparisons, and post hoc tests were followed by Fisher’s protected least significance difference (PLSD) tests for multiple pair-wise tests using Statview® statistical software (SAS institute Inc., USA).

## Results

### Gross findings and post-operative CT scan

No animal died due to complications from the surgery or due to anesthetic complications. Additionally, the healing processes for each of the animals were uneventful. A total of 30 implants were placed. Twenty-seven implants survived and 3 implants were found loose with no stability. Each one of these three implants belonged to each of the three individual groups in the same side of the used animal. No other complications like infection or foreign-body rejection were found.

### Fluorochrome bone labeling

**Fig. 3:**
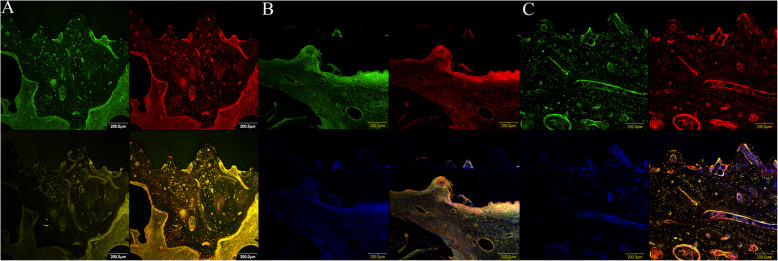
Confocal image showing the polychromic vital staining of Tetracycline HCl (3 and 11 weeks), Calcein (6 weeks), and Alizarin Red stain (9 weeks). No separable chronologic regenerative fluorescence bands could be observed in the three groups. **a** MSCs+PRP+PCL-TCP, **b** PRP+PCL-TCP, and **c** PCL-TCP groups (200-μm scale bar)

New bone formation was found between the implant and scaffold in the three groups**.** However, no separable chronologic regenerative fluorescence bands could be observed in the three groups (Fig. 3).

### Micro-CT bone morphogenic findings

**Fig. 4:**
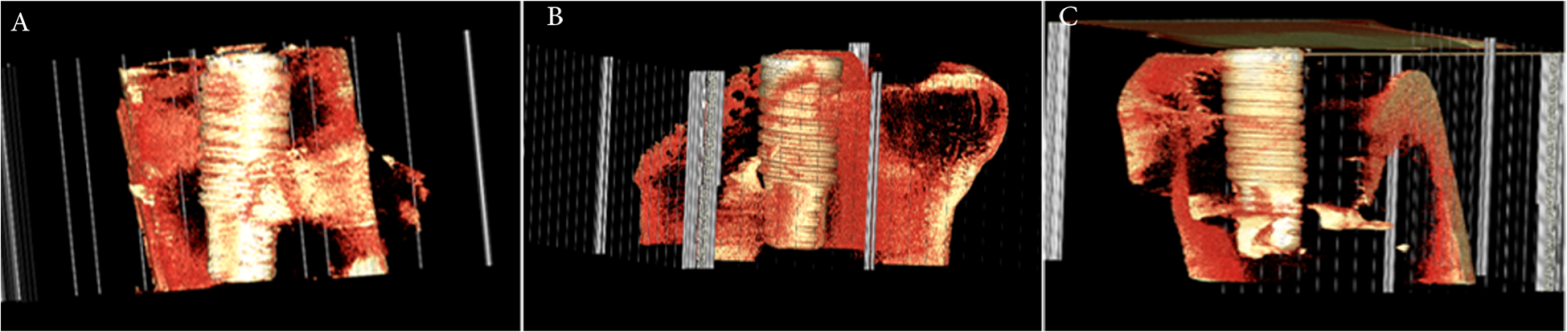
3D-rendered structure showing the MSCs+PRP+PCL-TCP group (**a**), the PRP+PCL-TCP group (**b**), and the PCL-TCP group (**c**) where more residual scaffold remnants can be observed compared to the other groups

The regenerative bone volume (BV) and bone volume fraction ratio (BV/TV) were non-significantly higher in the PCL-TCP group and MSCs+PRP+PCL-TCP group compared to the PRP+PCL-TCP group. The MSCs+PRP+PCL-TCP group showed non-significant higher bone surface (BS), bone surface density (BS/TV), and bone specific surface (BS/BV) than the other two groups (Fig. 4) (Table 1).

**Table 1:** Micro-CT bone morphogenic findings

Group	Number	Bone volume (BV, mm^**3**^)	Bone volume fraction ratio (BV/TV, %)	Bone surface (BS, mm^**2**^)	Bone surface density (BS/TV, mm^**−1**^)	Bone specific surface (BS/BV, mm^**−1**^)
MSCs+PRP+PCL-TCP	5	7.52 ± 4.12	8.64 ± 5.00	616.62 ± 212.39	7.14 ± 2.64	97.18 ± 36.47
PRP+PCL-TCP	5	6.96 ± 4.10	6.41 ± 3.87	522.20 ± 171.063	4.75 ± 1.53	97.05 ± 49.92
PCL-TCP	5	8.16 ± 5.80	10.18 ± 9.30	517.77 ± 255.075	5.76 ± 3.21	87.90 ± 48.38

*MSCs* mesenchymal stem cells, *PRP* platelet-rich plasma, *PCL-TCP* polycaprolactone (PCL)-β-tricalcium phosphate

### Histomorphometric findings

**Fig. 5:**
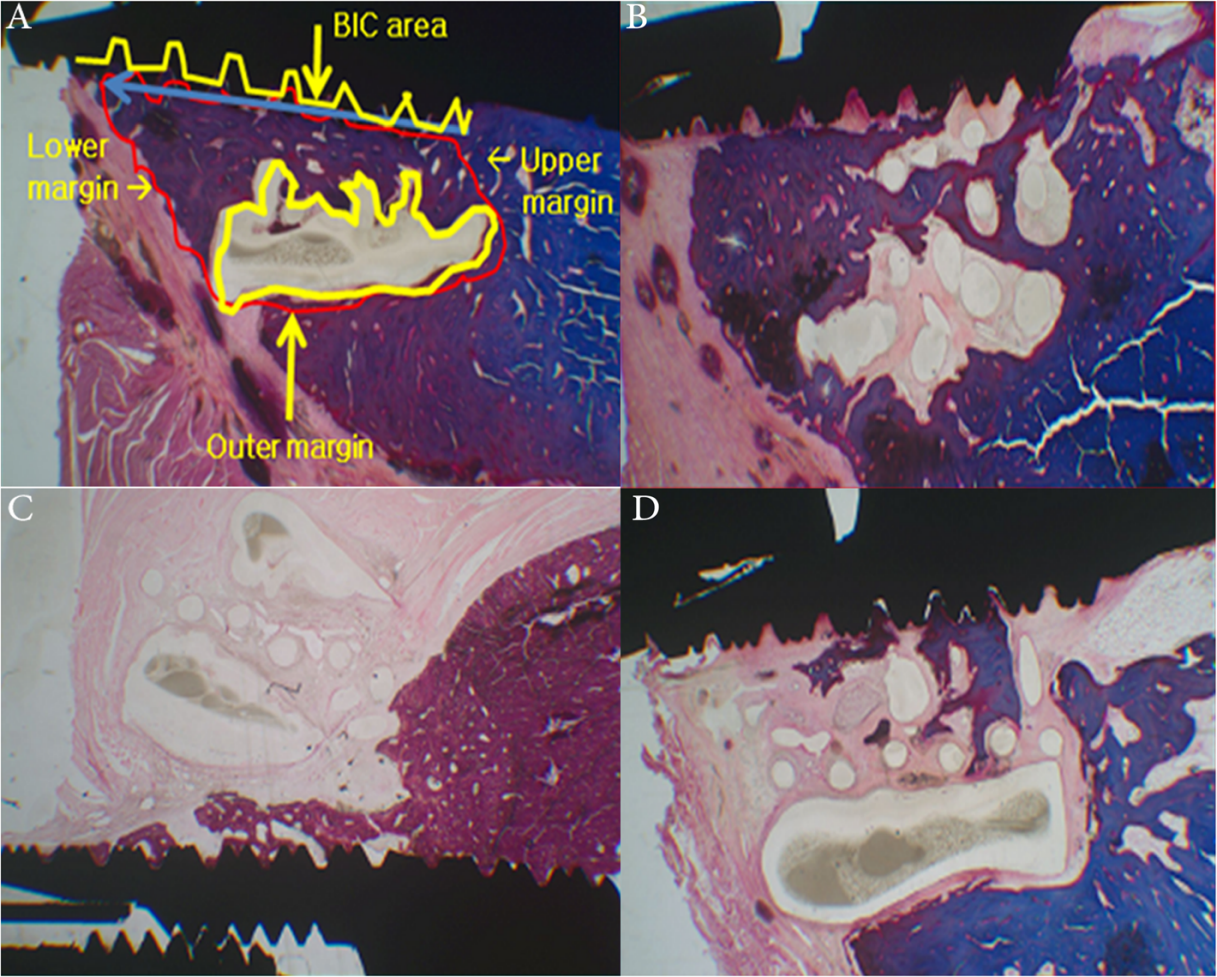
Histomorphometric analysis. **a** Parameters of histomorphometric analysis; new bone height (mm, blue line), new bone area (mm^2^, red polygonal area), bone-implant contact (red/yellow length ratio), and residual scaffold area (mm^2^, yellow polygonal area). **b**–**d** Microphotograph of the histological structure showing new regenerative bone along the implant contact surface with degradation of the scaffold at **b** MSCs+PRP+PCL-TCP, **c** PRP+PCL-TCP, and **d** PCL-TCP groups, respectively

The rate of scaffold degradation was slow in the PCL-TCP group and the PRP+PCL-TCP group compared to the MSCs+PRP+PCL-TCP group. Altogether, the MSCs+PRP+PCL-TCP group revealed higher bone-implant contact (BIC) ratio (*p* = 0.017) and new bone height (NBH) formation (*p* = 0.0097) with statistically significant difference in comparison with PCL-TCP group. The new bone area (NBA) was largely formed within the MSCs+PRP+Scaffold group, but with no statistically difference compared to the other groups (Fig. 5) (Table 2).

**Table 2:** Histomorphometric analysis

Group	***N***	New bone area (NBA, mm^**2**^)	New bone height (NBH, mm)	Bone-implant contact ratio (BIC)	Residual scaffold area (RSA, mm^**2**^)
MSCs+PRP+PCL-TCP	5	2.68 ± 0.90	3.55 ± 0.70*	4.07 ± 1.10**	3.38 ± 0.80
PRP+PCL-TCP	5	2.20 ± 0.80	2.65 ± 0.60	2.60 ± 0.70	4.50 ± 1.00
PCL-TCP	5	0.78 ± 0.30	1.31 ± 0.30	1.12 ± 0.50	6.06 ± 1.90

There was a significant statistical difference between the MSCs+PRP+PCL-TCP group and the PCL-TCP group, *p = 0.0097 and **p = 0.017

*MSCs* mesenchymal stem cells, *PRP* platelet-rich plasma, *PCL-TCP* polycaprolactone (PCL)-β-tricalcium phosphate

## Discussion

Many resorbable and non-resorbable membranes are used for the purpose of guided bone regeneration. Non-resorbable materials are frequently exposed throughout the soft tissue and need a second operation for removal [12]. Resorbable material like collagen is considered an outstanding biocompatible membrane. However, it lacks rigidity and susceptible to bone dehiscence and fenestration [13]. Polyesters like poly(ε) caprolactone (PCL) are characterized by manageability, biocompatibility, biodegradability, and rigidity. Poly(ε) caprolactone’s (PCL) main disadvantages are hydrophobicity, that lowers its cells affinity, and slow degradation rate, that may interfere with the process of bone regeneration [14, 15]. A controlled biodegradability is critical for providing the space gradually for bone regeneration. In the present study, β-tricalcium phosphate (TCP) was incorporated to improve the PCL scaffold stability, osteoconductivity, and increase the degradation rate.

Porosity is another important factor in the process of bone regeneration. Higher porosity is expected to improve bone formation by selectively increasing the diffusion of nutrients, oxygen, and removal of cellular waste products [16]. In this study, the scaffolds were designed with 70% porosity. A previous report showed that PCL/TCP membranes had a slightly higher elastic modulus and promoted the proliferation of pre-osteoblasts and inhibited fibroblast ingrowth in vitro accompanied with significantly higher bone regeneration values than PCL and collagen groups in vivo [17]. A better maintenance of the alveolar contour by PCL-TCP scaffold was also found in comparison with autogenous particulate bone within 6 months [18].

In the present study, the loading of MSCs+PRP into the PCL-TCP scaffold resulted in significant increase of new bone formation (new bone height) and better stability of implant(bone-implant contact ratio) compared to the mere PCL-TCP scaffold. This indicated the important role of MSCs in the guided bone regeneration. The results of the current study go in accordance with previous reports that showed improved bone regeneration after loading MSCs in the scaffolds [19, 20]. The scaffold is acting as a matrix to accommodate the cells to survive and subsequently enhance bone formation. MSCs are believed to enhance osteogenesis through immunosuppression effect. MSCs has the ability to reduce the level of tumor necrosis factor-α (TNF-α). TNF-α is known for its role in blockage of differentiation into osteoblasts [21]. In the present study, umbilical cord mesenchymal stem cells (UC- MSCs) were used. These types of stem cells possess longer culture period and higher proliferation capacity compared to bone marrow-derived MSCs [8]. MSCs can survive xenotransplantation without immune suppression and proved to be immunosuppressive. In one report, when pigs UC-MSCs were transplanted into rats, they did not stimulate immune rejection and survived for 6 weeks [22].

Platelet-rich plasma (PRP) enhanced the bone regeneration and degradation of the scaffold. PRP worked through release of growth factors like vascular endothelial growth factor (VEGF) which is primarily responsible for endothelial cells proliferation in angiogenesis. Angiogenesis or vascularization enhances bone regeneration by accelerating the differentiation and/or maturation of infiltrating osteoblasts and osteoblast precursor cells [7].

## Conclusion

The PCL-TCP scaffold proved to be compatible for bone regeneration in bone defects surrounding dental implants. Moreover, the incorporation of MSCs and PRP optimized the bone regeneration process with respect to the rate of scaffold replacement, the height of the regenerated bone, and implant stability.

## Data Availability

The datasets used or analyzed during the current study are available from the corresponding author on reasonable request.

## Abbreviations

PCL: Poly(ε) caprolactone
TCP: β-tricalcium phosphate
MSCs: Mesenchymal stem cells
PRP: Platelet-rich plasma
hUCMSCs: Human umbilical cord derived mesenchymal stem cells
TV: Tissue volume
BS: Bone surface
BV: Bone volume
BV/TV: Bone volume fraction ratio
BS: Regenerative new bone surface area
BS/BV: Bone specific surface
BS/TV: Bone surface density
I.S: Interception surface
NBH: New bone height
NBA: New bone area
BIC: Bone-implant contact
RSA: Residual scaffold areas

## References

[1] Kulakov AA, Gvetadze RS, Brailovskaya TV, Khar'kova AA, Dzikovitskaya LS (2017). Modern approaches to dental implants placement in deficient alveolar bone. Stomatologiia (Mosk).

[2] Widmark G, Andersson B, Ivanoff CJ (1997). Mandibular bone graft in the anterior maxilla for single-tooth implants. Presentation of surgical method. Int J Oral Maxillofac Surg..

[3] Silber JS, Anderson DG, Daffner SD, Brislin BT, Leland JM, Hilibrand AS, Vaccaro AR, Albert TJ (2003). Donor site morbidity after anterior iliac crest bone harvest for single-level anterior cervical discectomy and fusion. Spine (Phila Pa 1976)..

[4] Karring T, Nyman S, Gottlow J, Laurell L (1993). Development of the biological concept of guided tissue regeneration--animal and human studies. Periodontol 2000.

[5] Williams JM, Adewunmi A, Schek RM, Flanagan CL, Krebsbach PH, Feinberg SE, Hollister SJ, Das S (2005). Bone tissue engineering using polycaprolactone scaffolds fabricated via selective laser sintering. Biomaterials..

[6] Choong C, Triffitt J, Cui Z (2004). Polycaprolactone scaffolds for bone tissue engineering: effects of a calcium phosphate coating layer on osteogenic cells. Food Bioproducts Processing.

[7] Marx RE, Carlson ER, Eichstaedt RM, Schimmele SR, Strauss JE, Georgeff KR (1998). Platelet-rich plasma: growth factor enhancement for bone grafts. Oral Surg Oral Med Oral Pathol Oral Radiol Endod..

[8] Kern S, Eichler H, Stoeve J, Kluter H, Bieback K (2006). Comparative analysis of mesenchymal stem cells from bone marrow, umbilical cord blood, or adipose tissue. Stem Cells..

[9] Masuki H, Okudera T, Watanebe T, Suzuki M, Nishiyama K, Okudera H (2016). Growth factor and pro-inflammatory cytokine contents in platelet-rich plasma (PRP), plasma rich in growth factors (PRGF), advanced platelet-rich fibrin (A-PRF), and concentrated growth factors (CGF). Int J Implant Dentistry..

[10] Bilodeau K, Mantovani D (2006). Bioreactors for tissue engineering: focus on mechanical constraints. A comparative review. Tissue Eng..

[11] Song K, Liu T, Cui Z, Li X, Ma X (2008). Three-dimensional fabrication of engineered bone with human bio-derived bone scaffolds in a rotating wall vessel bioreactor. J Biomed Mater Res A..

[12] Chiapasco M, Zaniboni M (2009). Clinical outcomes of GBR procedures to correct peri-implant dehiscences and fenestrations: a systematic review. Clin Oral Implants Res..

[13] Dimitriou R, Mataliotakis GI, Calori GM, Giannoudis PV (2012). The role of barrier membranes for guided bone regeneration and restoration of large bone defects: current experimental and clinical evidence. BMC Med..

[14] Lam CX, Hutmacher DW, Schantz JT, Woodruff MA, Teoh SH (2009). Evaluation of polycaprolactone scaffold degradation for 6 months in vitro and in vivo. J Biomed Mater Res A..

[15] Domingos M, Intranuovo F, Gloria A, Gristina R, Ambrosio L, Bartolo PJ (2013). Improved osteoblast cell affinity on plasma-modified 3-D extruded PCL scaffolds. Acta Biomater..

[16] Hing KA (2005). Bioceramic gone braft substitutes: influence of porosity and chemistry. Int J Appl Ceramic Technol.

[17] Shim JH, Won JY, Park JH, Bae JH, Ahn G, Kim CH, et al. Effects of 3D-printed polycaprolactone/beta-tricalcium phosphate membranes on guided bone regeneration. Int J Mol Sci. 2017;18(5). 10.3390/ijms18050899 PMC5454812 28441338

[18] Goh BT, Chanchareonsook N, Tideman H, Teoh SH, Chow JK, Jansen JA (2014). The use of a polycaprolactone-tricalcium phosphate scaffold for bone regeneration of tooth socket facial wall defects and simultaneous immediate dental implant placement in Macaca fascicularis. J Biomed Mater Res A..

[19] Nuntanaranont T, Promboot T, Sutapreyasri S (2018). Effect of expanded bone marrow-derived osteoprogenitor cells seeded into polycaprolactone/tricalcium phosphate scaffolds in new bone regeneration of rabbit mandibular defects. J Mater Sci Mater Med..

[20] Lee JW, Chu SG, Kim HT, Choi KY, Oh EJ, Shim J-H, Yun WS, Huh J, Moon S, Kang S, Chung H (2017). Osteogenesis of adipose-derived and bone marrow stem cells with polycaprolactone/tricalcium phosphate and three-dimensional printing technology in a dog model of maxillary bone defects. Polymers..

[21] Kovach TK, Dighe AS, Lobo PI, Cui Q (2015). Interactions between MSCs and immune cells: implications for bone healing. J Immunol Res..

[22] Weiss ML, Mitchell KE, Hix JE, Medicetty S, El-Zarkouny SZ, Grieger D (2003). Transplantation of porcine umbilical cord matrix cells into the rat brain. Exp Neurol..

